# Cerebral Gluconeogenesis and Diseases

**DOI:** 10.3389/fphar.2016.00521

**Published:** 2017-01-04

**Authors:** James Yip, Xiaokun Geng, Jiamei Shen, Yuchuan Ding

**Affiliations:** ^1^Department of Neurosurgery, Wayne State University School of MedicineDetroit, MI, USA; ^2^China-America Institute of Neuroscience, Beijing Luhe Hospital, Capital Medical UniversityBeijing, China; ^3^Department of Neurology, Beijing Luhe Hospital, Capital Medical UniversityBeijing, China

**Keywords:** gluconeogenesis, glycolysis, stroke, glioma, metastatic breast cancer, tumor-infiltrating lymphocytes, lactate, pyruvate recycling

## Abstract

The gluconeogenesis pathway, which has been known to normally present in the liver, kidney, intestine, or muscle, has four irreversible steps catalyzed by the enzymes: pyruvate carboxylase, phosphoenolpyruvate carboxykinase, fructose 1,6-bisphosphatase, and glucose 6-phosphatase. Studies have also demonstrated evidence that gluconeogenesis exists in brain astrocytes but no convincing data have yet been found in neurons. Astrocytes exhibit significant 6-phosphofructo-2-kinase/fructose-2,6-bisphosphatase-3 activity, a key mechanism for regulating glycolysis and gluconeogenesis. Astrocytes are unique in that they use glycolysis to produce lactate, which is then shuttled into neurons and used as gluconeogenic precursors for reduction. This gluconeogenesis pathway found in astrocytes is becoming more recognized as an important alternative glucose source for neurons, specifically in ischemic stroke and brain tumor. Further studies are needed to discover how the gluconeogenesis pathway is controlled in the brain, which may lead to the development of therapeutic targets to control energy levels and cellular survival in ischemic stroke patients, or inhibit gluconeogenesis in brain tumors to promote malignant cell death and tumor regression. While there are extensive studies on the mechanisms of cerebral glycolysis in ischemic stroke and brain tumors, studies on cerebral gluconeogenesis are limited. Here, we review studies done to date regarding gluconeogenesis to evaluate whether this metabolic pathway is beneficial or detrimental to the brain under these pathological conditions.

## Gluconeogenesis pathway

**Figure 1:**
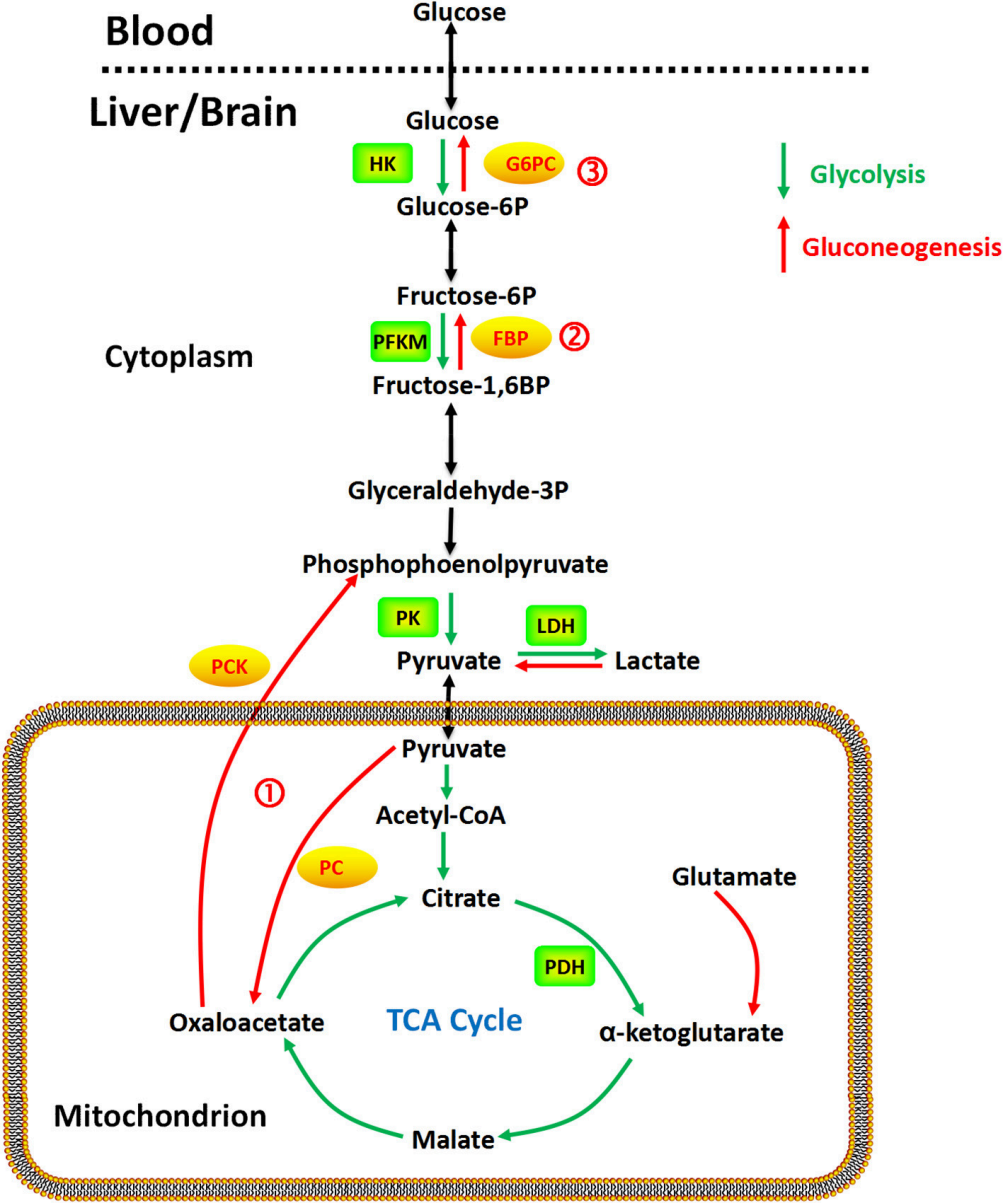
**Gluconeogenesis is a multistep metabolic process that generates glucose from pyruvate or a related three-carbon compound (lactate) and glutamine**. Several reversible steps in gluconeogenesis are catalyzed by the same enzymes used in glycolysis. There are three irreversible steps in the gluconeogenic pathway: (1) conversion of pyruvate to PEP via oxaloacetate, catalyzed by PC and PCK; (2) dephosphorylation of fructose 1,6-bisphosphate by FBP; and (3) dephosphorylation of glucose 6-phosphate by G6PC.

The gluconeogenesis pathway (Figure 1) has four irreversible steps catalyzed by the enzymes: pyruvate carboxylase (PC), phosphoenolpyruvate carboxykinase (PCK), fructose 1,6-bisphosphatase (FBP), and glucose 6-phosphatase (G6PC; van den Berghe, 1996), which have been found in the liver, kidney, intestine, and muscle. In the brain, astrocytes exhibit significant 6-phosphofructo-2-kinase/fructose-2,6-bisphosphatase-3 (PFKFB3) activity (Herrero-Mendez et al., 2009), a key mechanism for regulating glycolysis and gluconeogenesis through synthesis or hydrolysis of fructose-2,6-bisphosphate (Hers, 1983). Gluconeogenesis in astrocytes has been demonstrated with aspartate, glutamate, alanine, and lactate as precursors (Ide et al., 1969; Phillips and Coxon, 1975; Dringen et al., 1993a; Schmoll et al., 1995). Alterations in promoter methylation of the fructose 1,6-bisphosphatase gene, which is the rate limiting enzyme in the gluconeogenic pathway, have been found in cancer cells, potentially affecting mRNA levels and expression of the enzyme (Bigl et al., 2008). No studies have found evidence of gluconeogenic activity in neurons to our knowledge.

PC is a mitochondrial enzyme in the ligase class that catalyzes the irreversible carboxylation of pyruvate to oxaloacetate in the metabolic pathway of gluconeogenesis. The reaction is dependent on biotin, adenosine triphosphate (ATP) and magnesium (Jitrapakdee and Wallace, 1999; Jitrapakdee et al., 2008). Acetyl-coenzyme A (Acetyl-CoA) is the allosteric effector of PC in humans (Adina-Zada et al., 2012).

PCK is an enzyme in the lyase family that converts oxaloacetate into phosphoenolpyruvate and carbon dioxide, either in the cytosol or mitochondria via the cytosolic (PCK1) or mitochondrial (PCK2) isoforms of the enzyme, respectively. In the human liver, PCK is approximately equally distributed in the cytosol and the mitochondria (Atkin et al., 1979). Cytosolic PCK has been found with FBP in the liver, kidney, small intestine, stomach, adrenal gland, testis, and prostate. The co-localization of these two enzymes in these tissues suggest that gluconeogenesis may not be restricted to liver and kidney (Yánez et al., 2003).

FBP is a cytosolic enzyme that catalyzes the dephosphorylation of fructose 1,6-bisphosphate to fructose 6-phosphate and inorganic phosphate in gluconeogenesis and the Calvin cycle (Paksu et al., 2011). Two human isoforms of the enzyme have been reported in the liver and muscle (Adams et al., 1990). Both isoforms are inhibited by adenosine monophosphate (AMP) and fructose 2,6-bisphosphate, a competitive substrate inhibitor of fructose 1,6-bisphosphate (Dzugaj and Kochman, 1980; el-Maghrabi et al., 1993; Tillmann and Eschrich, 1998). FBP activity is upregulated by 1,25-dihydroxyvitamin D3 in normal monocytes (Fujisawa et al., 2000). The liver isoenzyme has also been found in the kidney, type II pneumocytes, and monocytes (Dzugaj and Kochman, 1980; Kikawa et al., 1994; Gizak et al., 2001). Human FBP has been detected in leukocytes (Sybirna et al., 2006), prostate, ovary, adrenal gland, pancreas, heart, and stomach (Yánez et al., 2003). FBP inhibitors are being investigated as potential therapy for type 2 diabetes due their capability to reduce gluconeogenesis (van Poelje et al., 2011).

G6PC is an enzyme situated in the endoplasmic reticulum and hydrolyzes glucose 6-phosphate to produce glucose and inorganic phosphate. A number of isoforms have been noted in humans, including glucose 6-phosphatase-α (G6PC), glucose 6-phosphatase-2 (G6PC2), and glucose 6-phosphatase-β (G6PC3; Hutton and O'Brien, 2009). In humans, the glucose 6-phosphatase-α (G6PC) gene is primarily expressed in the liver, kidney, intestine, and less so in pancreatic islets, although current knowledge on this gene's tissue expression and its enzyme characteristics is limited. The *g6pc2* gene is predominantly expressed in pancreatic islets (Hutton and O'Brien, 2009), whereas the g*6pc3* gene is ubiquitously expressed with predominance in the brain, muscle, and kidney (Martin et al., 2002).

The bifunctional 6-phosphofructo-2-kinase/fructose-2,6-bisphosphatase (PFKFB) is responsible for phosphorylating fructose 6-phosphate to fructose-2,6-bisphosphate, which in turn activates phosphofructokinase-1 and the glycolytic pathway (Yalcin et al., 2009). Of the four PFKFB isoenzymes, PFKFB3 is distinguished by the presence of multiple AUUUA instability motifs in its 3′ untranslated region (Chesney et al., 1999), a very high kinase-to-phosphatase activity ratio (740:1; Sakakibara et al., 1997), high expression in rapidly proliferating transformed cells (Chesney et al., 1999), solid tumors and leukemias (Chesney et al., 1999; Kessler and Eschrich, 2001; Atsumi et al., 2002), and regulation by several proteins essential for tumor progression [e.g., HIF-1α (Obach et al., 2004), Akt (Manes and El-Maghrabi, 2005), and PTEN (Cordero-Espinoza and Hagen, 2013)]. Different nomenclature also recognizes two PFKFB3 isoforms, termed “inducible” and “ubiquitous” (Navarro-Sabaté et al., 2001). The inducible isoform has been shown to be induced by hypoxia. Heterozygous genomic deletion of the *pfkfb3* gene has been found to reduce both the glucose metabolism and growth of tumors in mice (Telang et al., 2006).

Taken together, as shown in Figure 1, gluconeogenesis is a multistep metabolic process that generates glucose from pyruvate or a related three-carbon compound (lactate, alanine). Seven reversible steps in gluconeogenesis are catalyzed by the same enzymes used in glycolysis. There are three irreversible steps in the gluconeogenic pathway: (1) conversion of pyruvate to PEP via oxaloacetate, catalyzed by PC and PCK; (2) dephosphorylation of fructose 1,6-bisphosphate by FBP-1; and (3) dephosphorylation of glucose 6-phosphate by G6PC.

## Glycolysis and gluconeogenesis in the brain

It is commonly believed that gluconeogenesis is normally present only in the liver, kidney, intestine, or muscle (Chen et al., 2015). Emerging studies, however, are showing evidence that gluconeogenic activity can also occur in the brain. While initial studies were not able to detect dephosphorylation of glucose-6-phosphate (Nelson et al., 1985; Dienel et al., 1988; Schmidt et al., 1989), subsequent studies revealed a functional G6PC complex in the brain (Bell et al., 1993; Forsyth et al., 1993; Schmoll et al., 1997) capable of hydrolyzing glucose-6-phosphate into glucose at a significant rate (Ghosh et al., 2005). Immunofluorescence studies have shown co-localization of glial fibrillary acidic protein (GFAP) with G6PC in astrocytes. While reactive astrocytes in a variety of abnormal brains were strongly G6PC positive, neoplastic astrocytes were often only weakly positive. G6PC was yet found in radial glia, neurons or oligodendroglia. Normally, astrocytes store glycogen. The demonstration that a subset of astrocytes also contain G6PC suggests that they are competent in gluconeogenesis, serving as a potential energy pathway for neurons (Bell et al., 1993). It has been suggested that G6PC may be silent under physiological conditions and become activated at times of stress (Ghosh et al., 2005). It is also possible that G6PC is not an essential enzyme for astrocytes to release glucose, and instead use a glucose concentration gradient to promote flow of glucose from astrocytes to neurons (Gandhi et al., 2009).

The interstitial microenvironment in the brain is unique. Due to the metabolic gatekeeping of astrocytes, which form bridges between neurons and blood vessels, the interstitial space is characterized by low levels of glucose (Fellows et al., 1992), high levels of glutamate (Yudkoff et al., 1993), and high levels of branched chain α-ketoacids (Daikhin and Yudkoff, 2000). After passing through the blood–brain barrier (BBB), glucose is mainly taken up and processed by astrocytes for neuronal energy requirements (Pellerin, 2008), resulting in an interstitial glucose level that is lower than that in the blood (Fellows et al., 1992; Gruetter et al., 1992). Brain glutamate consists of amino groups primarily derived from branched chain amino acids (BCAA; (Yudkoff et al., 1993)). This is made possible by neutral amino acid transporters that are highly expressed in brain endothelial cells (del Amo et al., 2008). Astrocytes then produce glutamine via transfer of an amino group from BCAA to glutamate, derived from α-ketoglutarate through the TCA cycle, with the resulting branched chain α-ketoacids released into the interstitial space and taken up by neurons for glutamine metabolism by deamination (Yudkoff et al., 1993).

Astrocytes are unique in that they use glycolysis to produce lactate, which is then shuttled into neurons and used for oxidative metabolism as yet another source of energy (Dringen et al., 1993b). Excess lactic acid is either removed via the vasculature or temporarily stored by metabolic conversion into glucose and glycogen or into alanine (Dringen et al., 1993a). Signal transduction involved in glycogen synthase (GS) activation (Hurel et al., 1996; Sung et al., 1998) aids in lactic acid conversion to glycogen in astrocytes and other cells with gluconeogenic potential (Dringen et al., 1993a; Bernard-Hélary et al., 2002). At times of high energy demand, lactate is formed as byproducts of anaerobic glycolysis by neighboring neurons, which can subsequently be used as substrates for gluconeogenesis. By retaining lactate intracellularly, lethal levels of lactic acidosis can be prevented by the use of gluconeogenic processes in astrocytes (Beckner et al., 2005).

In the liver, pyruvate is produced within the cell cytoplasm from glucose via glycolysis or conversion of alanine via alanine aminotransferase (ALT) in the Cahill cycle, which is then transported into the mitochondria (Bricker et al., 2012). Within the mitochondria, pyruvate may act as a substrate for the pyruvate dehydrogenase (PDH) complex, via the oxidative pathway, to produce ATP through the tricarboxylic acid (TCA) cycle and the oxidative phosphorylation reaction, or it can be taken up by PC through the gluconeogenesis pathway to produce glucose. In oxidative phosphorylation, oxidation of pyruvate to carbon dioxide involves the collaboration of the PDH complex, the TCA cycle, and the mitochondrial respiratory chain, which consumes oxygen to produce energy in the form of ATP. Under hypoxia or oxidative phosphorylation enzyme dysfunction, mitochondrial ATP production becomes interrupted. Under these circumstances, glycolysis becomes the primary source of energy, increasing the generation of lactate, an anion produced by lactate dehydrogenase (LDH) in the last step of glycolysis. In addition, impairment of the rate-limiting enzyme (FBP) in gluconeogenesis also results in lactate accumulation, as this metabolic route represents the predominant pathway to lactate utilization.

Enzymes involved in lactate metabolism have been shown to play critical roles in cancer cell growth and survival. In patients with G6PC deficiency (von Gierke disease), a significant difference in the cerebral arterio-venous lactate concentration has been demonstrated, suggesting that lactate may be used as an energy source by the brain (Fernandes et al., 1982). In ischemic stroke, hypoxia causes accumulation of lactic acid intracellularly, resulting in inhibition of glycolysis and subsequent suppression of ATP production. Neither mitochondrial oxidative phosphorylation nor anaerobic glycolysis alone can produce ATP at a sufficient rate to maintain brain function (D'Alecy et al., 1986).

To date, other enzymes involved in the gluconeogenesis pathway, such as PC and PFKFB, have yet to be elucidated in the brain.

## Gluconeogenesis under pathological conditions

Gluconeogenesis has been found to play a role in several pathological conditions. In the setting of ischemic stroke, mitochondrial ATP production becomes interrupted. Glycolysis becomes the primary source of energy, increasing the generation of lactate. Glucagon, a peptide hormone that activates gluconeogenesis, has a stimulatory effect on brain mitochondrial oxidative phosphorylation and may play a role in neuroprotection against hypoxic damage (D'Alecy et al., 1986). High glutamate levels have been implicated to be neurotoxic in stroke, head trauma, multiple sclerosis, and neurodegenerative diseases (Matés et al., 2002). The brain interstitium also contains glutamine (Yudkoff et al., 1993) and BCAA (Yudkoff, 1997; Daikhin and Yudkoff, 2000), which can serve as energy substrates through gluconeogenesis (DeBerardinis et al., 2007) and contribute to brain cancer growth and survival. Glucose formed by hepatic gluconeogenesis may be metabolized in brain tumors and generate lactate through glycolysis (Pichumani et al., 2016). Gliomas with low levels of phosphorylated Akt have been demonstrated to respond to erlotinib (Haas-Kogan et al., 2005). While increased levels of glycolytic enzymes were found in brain cancer cells (Chen et al., 2007; Palmieri et al., 2009), enhanced glucose uptake is not a feature of breast cancer brain metastasis (Chen, 2007; Kitajima et al., 2008; Bochev et al., 2012; Manohar et al., 2013). Brain metastatic cancer cells from the breast proliferate in the absence of glucose by acquiring enhanced FBP-based gluconeogenesis capabilities (Chen et al., 2015). Furthermore, the high metabolic demand and nutrient consumption of tumor cells prevent tumor-infiltrating lymphocytes (TIL) proliferation and differentiation, leading to functional impairment through suppressed IFN-γ production (Chang et al., 2013; Gubser et al., 2013) and TIL exhaustion (Ho et al., 2015). Phosphoenolpyruvate deficiency was found to increase sarco/endoplasmic reticulum Ca^2+^-ATPase (SERCA)-mediated Ca^2+^ re-uptake, preventing Ca^2+^- nuclear factor of activated T cells (NFAT) signaling and T-cell activation (Ho et al., 2015). Promoting phosphoenolpyruvate production in T cells may prove to be a promising strategy to improve the tumoricidal effects of TIL and adoptive cellular transfer (ACT) (Ho et al., 2015). Other enzymes involved in the gluconeogenesis pathway, such as PC and PFKFB, have not been well-studied in the brain under pathological conditions.

### Ischemic stroke

Clinical experience and animal model studies have led to the conclusion that hypoxia initially begins with a compromise in brain function, followed by respiratory and then finally cardiovascular collapse. Lundy et al. (1984) have shown that hypoxic rats first lose brain electrical activity, have respiratory arrest ~84 s later, and then finally experience cardiovascular collapse after another 71 s. Studies done in hypoxic dogs have likewise found that brain electrical activity ceases before the animals experience cardiovascular collapse (Herin et al., 1978). Previous studies have demonstrated that elevated blood ketones increased survival times of up to five times longer in mice subjected to hypoxic conditions (Eiger et al., 1980). Similarly, butanediol-induced ketosis was associated with improved neurologic function in hypoxic rats, and exogenous glucagon further potentiated this hypoxic tolerance (Eiger et al., 1980).

Hyperglycemia during acute stress has been associated with increased mortality (Dungan et al., 2009). Glucose control improves clinical outcomes, particularly in hospitalized patients with acute myocardial infarctions, undergoing coronary bypass surgery, or patients on ventilator support (Furnary et al., 2003; Malmberg et al., 2005; Van den Berghe et al., 2006). A high proportion of patients with acute stroke may develop hyperglycemia, including those without pre-existing diabetes (Capes et al., 2000; Kent et al., 2001; McCormick et al., 2008). Multiple studies suggest that stress-induced hyperglycemia after acute stroke is associated with a high risk of morbidity and mortality (Capes et al., 2000; Kent et al., 2001; McCormick et al., 2008). Stress-induced hyperglycemia has been attributed to increased stressed hormones, increased autonomic outflow from the hypothalamus or medulla, unmasking of occult diabetes mellitus, decreased plasma insulin concentrations or organ sensitivity, or damage to the glucose-regulating centers in the brain (Wass and Lanier, 1996). The toxicity of hyperglycemia does not appear to be related to the osmotic load of glucose or the direct effect of lactate, but the increase in blood glucose concentrations at the time of brain ischemia provides more substrate for anaerobic glycolysis and worsening intracellular acidosis (Pulsinelli et al., 1982). The resulting acidosis interferes with glycolysis, protein synthesis and activity, ion homeostasis, neurotransmitter release and reuptake, enzyme function, free radical production or scavenging, and stimulus-response coupling (Wass and Lanier, 1996). Previous studies have supported the point of demarcation between good and poor outcomes for glucose concentrations ranging from ~100 to 400 mg/dL (Wass and Lanier, 1996). Interestingly, while glucose-mediated exacerbation of neurological injury is well-documented in adults, it may not occur in newborns (Vannucci and Yager, 1992). Glucose pretreatment in perinatal animals have been shown to prolong survival and decrease permanent brain damage after systemic hypoxia, asphyxia, or cerebral ischemia. These studies highlight the importance of stress hyperglycemia as a pathologic factor in stroke progression, and suggests that lowering blood glucose levels after ischemic stroke may improve clinical outcome.

Glucagon levels may be elevated in stress conditions such as hypoxia and starvation. Glucagon has a direct and substrate-specific stimulatory effect on brain mitochondrial oxidative phosphorylation and may play a role in neuroprotection against hypoxic damage by stimulating or sustaining mitochondrial ATP production necessary for neuronal function (D'Alecy et al., 1986). Plasma glucagon levels of 0.7 μg/ml have been observed in pathological conditions such as exsanguination (Lindsey et al., 1975). Investigators have shown that systemic administration of glucagon can stimulate oxidative phosphorylation in hepatic (Siess and Wieland, 1978) and cardiac cells (Friedmann et al., 1980). Kirsch et al. (Kirsch and D'Alecy, 1984) found that glucagon enhanced the incorporation of β-hydroxybutyrate into CO_2_ in rat brain slices. However, D'Alecy et al. found that glucagon's stimulatory effect on ATP production is not due to direct stimulation of β-hydroxybutyrate oxidation (D'Alecy et al., 1986). Glucagon's stimulatory effect on mitochondrial oxidative phosphorylation is thought to be mediated by adenylate cyclase activation, producing elevated cytosolic 3′,5′-cAMP and ultimately acting to stimulate electron flow between cytochrome c_1_ and cytochrome c (Garrison and Haynes, 1975; Halestrap, 1978; Hoosein and Gurd, 1984). Glucagon may also act directly on isolated mitochondria and specifically alter oxidative metabolism, as Yun J. et al. (2009) I-labeled monoiodoglucagon has been demonstrated to directly bind to rat brain membranes and mitochondria and alter glutamate-mediated oxidative metabolism (D'Alecy et al., 1986). Glucagon may confer neuroprotection by stimulating mitochondrial substrate oxidation and ATP production which had been initially suppressed by hypoxia.

Glutamine, a substrate used in gluconeogenesis, is a precursor molecule for glutathione, which protects against ROS toxicity. It has been shown that glutamine supplementation can maintain high levels of glutathione and subsequently avoid oxidative stress damage (Amores-Sánchez and Medina, 1999). However, on the other spectrum, high glutamate levels has been implicated to be neurotoxic in stroke, head trauma, multiple sclerosis and neurodegenerative diseases (Matés et al., 2002). It has been shown that exogenous α-tocopherol could prevent *N*-methyl-d-aspartate (NMDA)-induced increases in glutamine synthetase, an enzyme specific to glial cells. As α-tocopherol is an antioxidant, its involvement suggests that ROS may be associated with the glutamate excitotoxic process (Davenport Jones et al., 1998).

Hepatic gluconeogenesis activity has been demonstrated in rat models to be significantly increased in the setting of cerebral ischemia (Wang et al., 2013). In the acute phase (24 h) of stroke, rats developed higher fasting blood glucose and insulin levels in addition to the upregulation of hepatic gluconeogenic gene expression, including phosphoenolpyruvate carboxykinase, glucose-6-phosphatase, and fructose-1,6-bisphosphatase (Wang et al., 2013). Hepatic gluconeogenesis-associated positive regulators, such as FoxO1, CAATT/enhancer-binding proteins (C/EBPs), and cAMP responsive element-binding protein (CREB), were also upregulated. In terms of insulin signaling transduction, the phosphorylation of insulin receptor (IR), insulin receptor substrate-1 (IRS1) at the tyrosine residue, Akt, and AMP-activated protein kinase (AMPK), were attenuated in the liver, while negative regulators such as phosphorylation of p38, c-Jun N-terminal kinase (JNK), and IRS1 at the serine residue, were increased. In addition, the brains of rats with stroke exhibited a reduction in phosphorylation of IRS1 at the tyrosine residue and Akt. Circulating cortisol, glucagon, C-reactive protein (CRP), monocyte chemoattractant protein 1 (MCP-1), and resistin levels were elevated, but adiponectin was reduced. This suggests that cerebral ischemic stroke may modify the intracellular and extracellular environments, favoring hyperglycemia, and hepatic gluconeogenesis.

### Gliomas

One of the mechanisms for cancer cell growth and survival is enhanced glucose metabolism through aerobic glycolysis, also known as the Warburg effect (Vander Heiden et al., 2009). There is a high metabolic demand in malignant tumor cells for biochemical building blocks, such as amino acids for protein synthesis, nucleic acids for gene replication, and fatty acids for phospholipid membrane barriers (Locasale and Cantley, 2011). Amino acids, such as glutamine, has been shown to be a source of energy production in gluconeogenesis (DeBerardinis et al., 2007). In advanced-stage cancers, energy may be derived from enhanced oxidation of BCAA, valine, leucine, and isoleucine (Beck and Tisdale, 1989; Pisters and Pearlstone, 1993; Baracos and Mackenzie, 2006). The brain interstitium contains high levels of glutamine (Yudkoff et al., 1993) and BCAA (Yudkoff, 1997; Daikhin and Yudkoff, 2000) which can serve as energy substrates through gluconeogenesis (DeBerardinis et al., 2007), and their abundance may contribute to brain cancer growth and survival.

A number of evidence has demonstrated that cancer consists of a subset of stem cells that may be responsible for resistance to conventional cancer therapies and promote tumor growth (Hanahan and Weinberg, 2011). It has been observed that cancer stem cells from glioblastomas depend on G6PC and use the enzyme to counteract glycolytic inhibition (Abbadi et al., 2014). Interestingly, the knockdown of G6PC was able to decrease the aggressive phenotype of glioblastoma stem cells, potentially through the downregulation of the CD133/AKT pathway and an increase in glycogen accumulation through activation of GS and inhibition of glycogen phosphorylase, which has been previously shown to induce cancer cell death (Lee et al., 2004; Favaro et al., 2012). G6PC knockdown also reduced migration, invasion, and cell viability (Abbadi et al., 2014). A number of studies have suggested that cancer cells have elevated levels of glycogen (Rousset et al., 1981), which is accumulated in response to hypoxic stimulation for later use in several cancer cell lines (Pelletier et al., 2012). In U87 glioma cells, glycogen accumulation induces premature cell senescence (Favaro et al., 2012).

Recent studies have found that glioblastomas and brain metastases have the capacity to oxidize acetate in the citric acid cycle (Mashimo et al., 2014), which is unexpected as there is no simple pathway for acetate to enter the lactate or pyruvate pool (Cerdan et al., 1990; Håberg et al., 1998a,b; Deelchand et al., 2009; Marin-Valencia et al., 2012). There have been studies describing “pyruvate recycling,” where acetate converts into the TCA intermediates to generate pyruvate (Cerdan et al., 1990; Cruz et al., 1998; Håberg et al., 1998a,b; Serres et al., 2007; Deelchand et al., 2009). The net synthesis of pyruvate can be achieved by malate decarboxylation to pyruvate through the activity of malic enzyme, and oxaloacetate decarboxylation through the conjugated actions of PCK and pyruvate kinase (Olstad et al., 2007). Pyruvate can then enter the TCA cycle via acetyl-CoA. Pyruvate recycling is well-described in the liver (Freidmann et al., 1971) and the kidney (Rognstad and Katz, 1972). Pyruvate recycling degrades compounds such as glutamate, glutamine, or aspartate, which are originally derived from pyruvate carboxylation, to pyruvate and reenter the TCA cycle as acetyl CoA (Olstad et al., 2007). Pyruvate recycling has been reported in rat brain following infusion of acetate (Cerdan et al., 1990; Cruz et al., 1998).

In the liver, systemic acetate may also enter the citric acid cycle. Although net synthesis of glucose from acetyl groups does not occur in mammalian liver, acetate may convert into oxaloacetate and enter gluconeogenesis. Glucose formed by hepatic gluconeogenesis may then be metabolized in brain tumors and generate lactate through glycolysis, contributing to the brain tumor lactate pool (Pichumani et al., 2016). Studies that tracked radioactively-labeled acetate revealed that the majority of lactate in brain tumors is from acetate directly metabolized in human glioblastomas and brain metastasis, contributing up to 48% of the acetyl-CoA pool (Mashimo et al., 2014; Pichumani et al., 2016). Acetate may also produce lactate elsewhere in the body and enter blood circulation to be transported to the tumor and used as an energy source. Many human tumors have elevated lactate dehydrogenase 5 (LDH5) levels, and the lactate dehydrogenase C (LDHC) gene has been found to be expressed in many tumors. Alternatively, neighboring astrocytes may convert the monocarboxylate chain of lactate to glycogen and transport to neurons as glucose (DiNuzzo et al., 2011).

Mutations in the PTEN gene have also been demonstrated to commonly occur in gliomas (Cantley and Neel, 1999; Sano et al., 1999; Zundel et al., 2000; Fan et al., 2002), leading to loss of negative regulation on the phosphatidylinositol-3 kinase (PI3K)/Akt pathway. This results in phosphorylation, and hence deactivation, of GS kinase-3 (GSK3) and subsequent dephosphorylation/activation of GS. When the pathway is stimulated, inactivated GSK3 is unable to interact with other kinases to constitutively inhibit GS. Decreased expression of PTEN was found in 29 of 42 (69%) of glioblastomas from human patients based on immunostains (Sano et al., 1999). A study that tested six glioblastoma specimens through immunoblotting found decreased levels of PTEN in all six samples and increased activation/phosphorylation of downstream Akt in 4 of 6 (67%) glioblastomas (Ermoian et al., 2002). Recently, phosphorylated Akt has been found in 18 of 29 (62%) glioblastoma specimens and 22 of 40 (55%) gliomas of any grade. Interestingly, none of the 22 gliomas with high levels of phosphorylated Akt responded to treatment with erlotinib, an epidermal growth factor tyrosine kinase inhibitor. However, 8 of 18 tumors with low levels of phosphorylated Akt respond to the drug. Increased activation of the PI3K/Akt pathway was also associated with tumor progression in these specimens (Haas-Kogan et al., 2005).

Other factors include PDH, a potential mediator that protects against cancer and has been observed to reduce glioblastoma growth (Adeva et al., 2013). Mitochondrial DNA mutations have also been detected in a number of cancers. Succinate dehydrogenase genes have been shown to act as a tumor suppressor and thus mutations in these genes increases the risk of tumor progression (Adeva et al., 2013).

### Brain metastatic cancer (breast cancer)

One of the driving forces behind altered energy metabolism is the factors that influence the extrinsic tissue microenvironment, such as the presence of hypoxia or hypoglycemia (Yun J. et al., 2009). These factors exist in the microenvironment during unregulated tumor expansion and to which metastatic cancer cells migrate, which may contrast the primary site where nutrients and growth factors may be more abundant (Fidler, 2003; Martinez-Outschoorn et al., 2011). There is great diversity between the microenvironments of various tissues. Cancer cells can extravasate from their primary site and reach multiple organs, but its proliferation is restricted by the secondary site's microenvironment (Fidler, 2003). The malignancy of such cancer cells is largely determined by its compatibility with the microenvironment of the host tissue. Studies have shown that tissue stromal cells can be reprogrammed to metabolize lactate secreted by cancer cells (Martinez-Outschoorn et al., 2011; Yuneva et al., 2012).

The role of various energy sources in the growth and survival of metastatic brain cancer remains to be elucidated. It has been demonstrated that mRNA of genes involved in glycolysis are elevated in brain metastatic cells (Chen et al., 2007). However, with the low glucose level in the brain's interstitium, metastatic cancer growth, and survival would require metabolic reprogramming within cancer cells, such as enhancing gluconeogenic enzyme levels, or modifications in the tissue microenvironment to take advantage of other energy sources. While increased levels of glycolytic enzymes were found in brain cancer cells (Chen et al., 2007; Palmieri et al., 2009), studies have demonstrated that enhanced glucose uptake is not a feature of breast cancer brain metastasis (Chen, 2007; Kitajima et al., 2008; Bochev et al., 2012; Manohar et al., 2013). This suggests that glucose may not be the primary or only energy source for brain metastasis.

Recently, it was found that, unlike native brain cancer cells, brain metastatic cancer cells from the breast could proliferate in the absence of glucose by acquiring enhanced gluconeogenesis capabilities, with increased oxidation of BCAA and glutamine, and upregulation of FBP (Chen et al., 2015). The study also found FBP upregulation in clinical specimens of brain metastasis and growth inhibition when FBP is knocked down in orthotopic brain metastasis formed by breast cancer cells, suggesting that activation of FBP-based gluconeogenesis is important for the growth and survival of metastatic cancer cells in the brain. The role of BCAA in metastatic brain cancer survival is further supported by studies that found higher sensitivity in tracing (Adina-Zada et al., 2012) C-BCAA for brain metastasis imaging compared to the glucose analog tracer (Tillmann and Eschrich, 1998) FDG, suggesting high levels of BCAA uptake by brain metastatic cancer cells (Chen, 2007; Kitajima et al., 2008; Bochev et al., 2012; Manohar et al., 2013).

Interestingly, in hepatocellular carcinoma, glycolytic tumors with increased (Tillmann and Eschrich, 1998) F-FDG uptake use glucose as a nutrient source for proliferation, whereas low glycolytic tumors show increased (Adina-Zada et al., 2012) C-acetate uptake accompanying lipid synthesis (Vavere et al., 2008). This has been well-correlated to histological grade, with glycolytic cancers having a higher histological grade than low glycolytic tumors (Yun M. et al., 2009). In contrast to typical glycolytic tumors, low glycolytic tumors were still able to preserve hepatic gluconeogenesis with autophagy as a supporting mechanism (Jeon et al., 2015). With the advent of modern imaging techniques such as PET, radiolabeling of glucose with (Tillmann and Eschrich, 1998) F has successfully imaged the altered metabolism of cancer, revolutionizing conventional cancer diagnosis (Xu et al., 2013).

### Host-mediated immunity in malignancy

Tumor-infiltrating lymphocytes such as cytotoxic T cells are well-known to provide host protection against cancerous cells and infectious pathogens (Shiao et al., 2011; Braumüller et al., 2013). In tumors, however, the cytotoxic functions of TIL such as IFN-γ, IL-2, IL-17, and granzyme B production are inhibited by multiple environmental factors (Cham et al., 2008; Mellman et al., 2011; Michalek et al., 2011; Shiao et al., 2011; Finlay et al., 2012; Chang et al., 2013). Alterations in nutrient availability, such as lactate and tryptophan metabolites, in the tumor microenvironment can limit TIL activity (Yang et al., 2013). Increased expression of inhibitory checkpoint receptors, such as programmed cell death protein 1 (PD-1), lymphocyte-activation gene 3 (Lag3), and cytotoxic T-lymphocyte-associated protein 4 (CTLA-4) desensitizes T cell receptor (TCR) signaling and contributes to their functional impairment (Baitsch et al., 2012), commonly referred to as “functional exhaustion” (Wherry, 2011). These discoveries have led to the development of cancer immunotherapies that reawaken exhausted TIL by blocking inhibitory checkpoint receptors and the use of ACT with tumor-specific T cells to restore the repertoire of cytotoxic T cells to eradicate tumors.

T cells undergo a metabolic switch similar to cancer cells and upregulate aerobic glycolysis and glutaminolysis for proliferation and differentiation into activated effector T cells (Ho et al., 2015). PI3K, Akt, and mTOR activation triggers the switch to anabolic metabolism by inducing transcription factors such as Myc and hypoxia-inducible factor 1 (HIF1; Wang et al., 2011; MacIver et al., 2013). Anergic T cells are unable to activate Ca^2+^ and NFAT signaling and have diminished rates of aerobic glycolysis and anabolic metabolism following stimulation (Srinivasan and Frauwirth, 2007; Zheng et al., 2009). Similarly, CD8+ T cells with increased PD-1 expression are unable to activate mTOR or aerobic glycolysis following TCR stimulation, whereas T cells with hyper-HIF1α activity and aerobic glycolysis are refractory to functional exhaustion (Parry et al., 2005; Doedens et al., 2013; Staron et al., 2014).

It is likely that, given their similar metabolic profiles and nutrient requirements, the high metabolic demand and nutrient consumption of tumor cells prevent TIL proliferation and differentiation, leading to functional impairment. Recent studies have shown that when glycolytic rates are low, glyceraldehyde phosphate dehydrogenase (GAPDH) suppresses IFN-γ production in T cells (Chang et al., 2013; Gubser et al., 2013). Studies have also found that CD4+ T cells in tumors were deprived of glucose which resulted in diminished tumoricidal functions, suggesting that glucose deprivation might contribute to TIL exhaustion (Ho et al., 2015). Ho et al. (2015) also demonstrated increased hexokinase 2 (HK2) expression in melanoma cells that allowed for a more efficient evasion of CD4 T cell-mediated immune surveillance, indicating that competition for nutrients could exist between TIL and tumor cells. Furthermore, phosphoenolpyruvate deficiency was found to increase SERCA-mediated Ca^2+^ re-uptake, preventing Ca^2+^-NFAT signaling and T cell activation. Promoting phosphoenolpyruvate production in T cells may prove to be a promising strategy to improve the tumoricidal effects of TIL and ACT.

## Conclusion and future studies

In addition to glycolysis which has been extensively studied on the mechanisms of ischemic stroke and brain tumors, studies on alternative pathways, gluconeogenesis, during such a stress conditions, are limited. It is becoming more recognized as an important pathway for alternative energy sources in the brain.

The biochemical mechanisms for astrocytes to convert from glycolysis or glycogenolysis to gluconeogenesis for neuronal energy remain to be elucidated. AMP or hexose phosphate depletion may activate FBP and suppress phosphofructokinase. A decrease in the level of fructose-2,6-biphosphate by low phosphofructokinase activity may favor lactate or glutamate for oxidative energy production and glycogen synthesis. Further studies are needed to discover how the gluconeogenesis pathway is controlled in the brain, which may lead to the development of therapeutic targets to control energy levels, and therefore cellular survival, in ischemic stroke patients or inhibit gluconeogenesis in brain tumors to promote malignant cell death and tumor regression.

## Author contributions

JY participated in the study design, acquisition of data, interpretation of data, drafting and revising version to be published. XG participated in the critical revision and final approval of the version to be published. JS participated in the figure design of the version to be published. YD participated in the concept and study design, critical revision, and final approval of version to be published.

### Conflict of interest statement

The authors declare that the research was conducted in the absence of any commercial or financial relationships that could be construed as a potential conflict of interest.
